# Effects of the Fukushima Daiichi nuclear accident on goshawk reproduction

**DOI:** 10.1038/srep09405

**Published:** 2015-03-24

**Authors:** Kaori Murase, Joe Murase, Reiko Horie, Koichi Endo

**Affiliations:** 1 Graduate School of Natural Science, and Research Center for biological diversity, Nagoya City University, 1 Yamanohata, Mizuho-cho, Mizuho-ku, Nagoya, Aichi 467-8501, Japan; 2 Goshawk Protection Fund, 2-5-1 Hanawada, Utsunomiya, Tochigi 320-0027, Japan

## Abstract

Although the influence of nuclear accidents on the reproduction of top predators has not been investigated, it is important that we identify the effects of such accidents because humans are also top predators. We conducted field observation for 22 years and analysed the reproductive performance of the goshawk (*Accipiter gentilis fujiyamae*), a top avian predator in the North Kanto area of Japan, before and after the accidents at the Fukushima Daiichi nuclear power plant that occurred in 2011. The reproductive performance declined markedly compared with the pre-accident years and progressively decreased for the three post-accident study years. Moreover, it was suggested that these declines were primarily caused by an increase in the air dose rate of radio-active contaminants measured under the nests caused by the nuclear accidents, rather than by other factors. We consider the trends in the changes of the reproductive success rates and suggest that internal exposure may play an important role in the reproductive performance of the goshawk, as well as external exposure.

The goshawk (*Accipiter gentilis*) is a raptor and a top predator that preys on birds and mammals^1^,^2^. Although there have been some reports on the reproductive performance of small birds after the Chernobyl nuclear power plant disaster^3^,^4^,^5^,^6^, detailed investigations on raptors, such as the goshawk, have not been conducted. Understanding the impact of nuclear accidents on the reproductive performance of a predatory raptor will be significant for humans and other top predators. The predatory raptor is one of the most suitable species to estimate the effect of the radiation from nuclear accidents on the top predators, for such animal species cannot be said to suffer from economical or psychological issues, which are often disputed in human subjects^7^,^8^.

Previous studies have compared the characteristics of birds in areas with different degrees of radioactive contamination^4^,^5^,^9^,^10^. However, there is no existing research providing a detailed comparison of the reproductive performance of the same species at the same nest sites before and after a nuclear power plant disaster. We investigated the reproductive performance of the goshawk (*Accipiter gentilis fujiyamae*) in Japan, since 1992, before the 2011 Tōhoku earthquake and the accidents at the Fukushima Daiichi nuclear power plant. We mapped their breeding sites as part of our field research, and in this study, we compared their reproductive performance in the 19 years before the nuclear accidents with the three years after the accidents. Therefore, this study will provide relevant ecological information for discussing the impacts of a nuclear accident.

The previous studies that investigated the effects of a nuclear accident on wild birds reported abnormalities of body form and of reproductive performance, but the causes were not clearly identified. In addition, when a negative effect was observed, the cause may not have originated from the nuclear accident. Other factors, such as the environment near the breeding site (e.g., lack of food resources), can negatively affect the reproductive performance. In this study, we measured the air dose rates of radio-active contaminants under the nests and analysed the nest success^11^ with a Bayesian method, incorporating environmental factors into the statistical model.

In this paper, we show three results. The first is when the nuclear accidents began to affect the reproductive performance of the Goshawk, and which breeding stage was affected. The second is the relationship between the air dose rate under the nests and the Goshawk's reproductive performance. The third is the comparison between the effect of the air dose rate and the other environmental factors (e.g., canopy closure, micro-climate stability, the abundance or availability of prey species, predation, and human disturbance^11^) on the reproductive performance.

## Results

### Study area and observations

**Figure 1:**
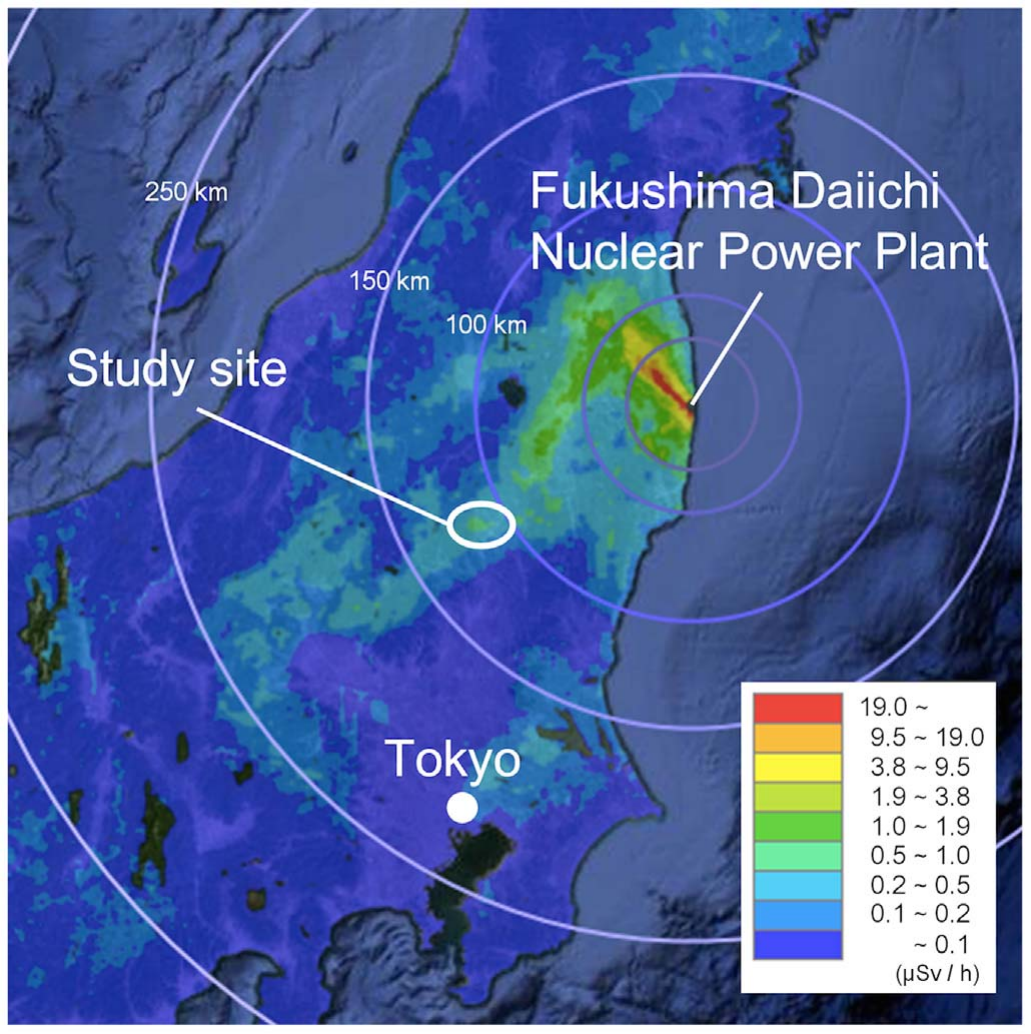
Study area. Our study area is located in North Kanto, Japan. The area is roughly indicated by the white circle in the figure for the protection of the goshawk. The Fukushima Daiichi nuclear power plant is within the range of 100 to 120 km from this area. Air dose rates (μSv/h) on May 31, 2012, are shown. Adapted from ‘Extension Site of Distribution Map of Radiation Dose, etc.,/Digital Japan’ (http://ramap.jmc.or.jp/map/eng/).

Our study area is shown in Figure 1. The goshawk is a protected bird species in Japan (Act on Conservation of Endangered Species of Wild Fauna and Flora; 4^th^ Red List, Near Threatened). To prevent illegal capture, the locations of their breeding sites are not shown. Goshawk reproductive performance at the breeding sites in 1992–2010 (prequake years) and 2011–2013 (postquake years) is shown in Tables 1 and 2. The air dose rates under the nests at 13 sites were measured in June 2012. The 13 nest sites were chosen at random. For these 13 sites, the air dose rates and the reproductive performances of goshawks for the period are shown in Table 3.

### Reproductive performance

**Figure 2:**
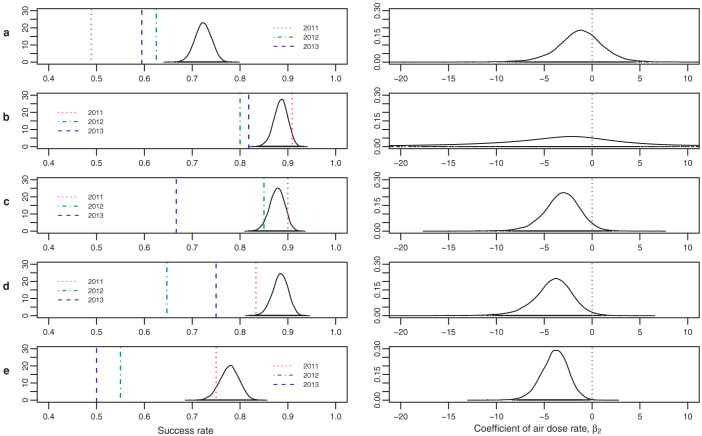
Success rates and effects of the air dose rate. The rates of the four breeding stages and the nest success (left) and the coefficients of the air dose rate corresponding to the stages (right) are shown. Posterior distributions of prequake rates are indicated as solid lines, and the rates of the postquake years are indicated as red dotted lines (2011), green dot-dashed lines (2012), and blue dashed lines (2013) (left). Posterior distributions of the air dose rate coefficients, *β*_2_, are indicated as solid lines (right). (a), Site occupancy (stage 1). (b), Incubating (stage 2). (c), Hatching (stage 3). (d), Fledging (stage 4). (e), Nest success.

The rates of the four breeding stages of the goshawk are shown in Table 4 and Figure 2, left. The four stages are site occupancy (stage 1), incubating (stage 2), hatching (stage 3), and fledging (stage 4). The rate of each postquake year was compared with the corresponding 95% credible interval (CRI) of the rate of the prequake 19 years. The nest success^11^, a proportion of fledged nests to incubated nests, are also shown (Fig. 2e, left, Table 4, Nest success).

In 2011, the site occupancy rate (stage 1) and fledging rate (stage 4) declined beyond the 95% CRIs of the prequake years (Fig. 2, left, Table 4). In 2012, the site occupancy rate (stage 1), incubating rate (stage 2), fledging rate (stage 4), and the nest success declined (Fig. 2, left, Table 4). In 2013, the rates of all the four stages and the nest success were outside the 95% CRIs (Fig. 2, left, Table 4).

### Influence of the air dose rate

We estimated the effect of the air dose rate on the breeding success. The measurements of the air dose rate were used with the observed data (Table 3). Using the air dose rate as an explanatory variable, a hierarchical Bayesian model accounting for differences among the breeding sites was employed (see Methods). The effect was estimated as a posterior distribution of *β*_2_, a coefficient of the air dose rate in our model, for each breeding stage. For the rates of the four stages and the nest success, the results are shown in Tables 4, 5, and Figure 2. For site occupancy (stage 1) and incubating (stage 2), the 95% CRIs contained zero near the centre (Figs. 2a and b, right, Table 5). In contrast, although for hatching (stage 3) the 95% CRI contained zero, the zero was on its upper boundary, and more than 96% of the posterior distribution had a negative value (Fig. 2c, right, Table 5). Moreover, for fledging (stage 4), the 95% CRI did not contain zero and more than 98% of the posterior distribution had a negative value (Fig. 2d, right, Table 5). The nest success also had a negative value in more than 99% of the posterior distribution (Fig. 2e, right, Table 5). These results indicated that the air dose rate under the nests did not associate with site occupancy (stage 1) or incubating (stage 2), whereas hatching (stage 3) and fledging (stage 4) were negatively associated with the air dose rate. Overall, the nest success was negatively and strongly associated with the air dose rate.

We described the characteristics of each stage by comparing its rate (Fig. 2, left) with the effect of the air dose rate (Fig. 2, right). For site occupancy (stage 1), although the air dose rate was not related to the early stages of breeding behaviour of the goshawk (Fig. 2a, right), the rate markedly declined (Fig. 2a, left). One likely explanation for the decline in rate is the presence of other environmental factors such as aftershocks.

For incubating (stage 2), although the air dose rate under the nests did not significantly associate with the incubating rate (Fig. 2b, right), the rate fell in 2012 and 2013 (Fig. 2b, left). This result may have been caused by the birds' life history during the nonbreeding season. In our study area, the goshawks hunt in a broader area in the nonbreeding season than in the breeding season^12^. It is likely that the condition of adult birds (particularly the egg-laying rate) was most closely related to the radioactive contamination of a broad circumferential area (i.e., the average radioactive level) and not with the air dose rate of the smaller breeding area. According to the official governmental report^13^, the area was contaminated mainly with radiocesium and other radionuclides. The decrease in the incubating rate in this study may be related to the accidents at the Fukushima Daiichi nuclear power plant. It is possible that the rate did not fall in 2011 because the nuclear accidents occurred in March, at the beginning of the goshawk breeding season: the adult goshawks in March 2011 would have been as healthy as during prequake years.

For hatching (stage 3), the air dose rates under the nests had negative association with the hatching rates (Fig. 2c, right), which dropped in 2013 (Fig. 2c, left). Moreover, for fledging (stage 4), the air dose rates under the nests had strong negative relationship with the fledging rates (Fig. 2d, right), which declined in all of the postquake years (Fig. 2d, left). Therefore, we suggest that the hatching rate and fledging rate of the goshawks was strongly related to the level of radioactive contamination under the nests. It is interesting that the rates of these stages tended to progressively decrease for years. One possible explanation is internal exposure (see Discussion).

The nest success (fledged/incubated) also clearly negatively associated with the air dose rate (Fig. 2e, right), and fell in 2012 and 2013 (Fig. 2e, left). Before the accidents at the Fukushima Daiichi nuclear power plant in 2011, the nest success of the goshawks did not fall as low as 50% as in 2013. The minimum value of the prequake years was 68% in 1992 (Table 1). Developing embryos or young individuals are strongly affected by radioactive contamination^14^, and the nestling goshawks are no exception.

### Air dose rate effect vs. site effect

**Figure 3:**
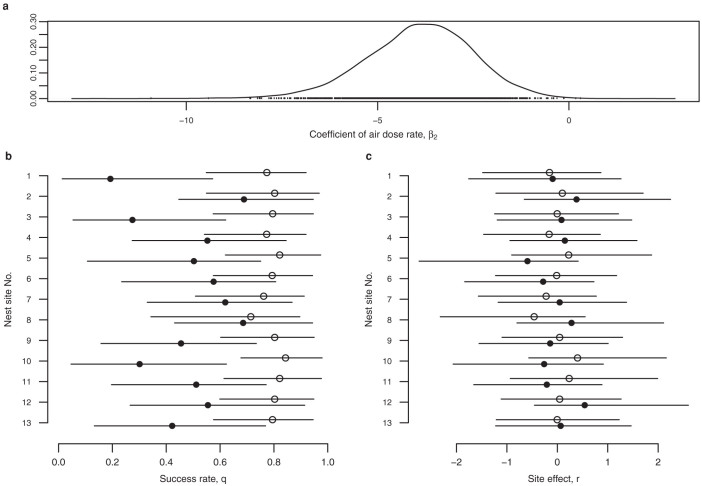
Results of the Bayesian analysis for the nest success. The posterior distributions of the parameters of the hierarchical Bayesian model for the nest success are shown. (a), The air dose rate coefficient, *β*_2_. This is the same figure as Figure 2e, right, except for its range. (b), The nest success of each breeding site. (c), Site effect of each breeding site. The open circles indicate prequake nest success, and the filled circles indicate postquake nest success. The horizontal bars indicate 95% CRIs.

To clearly show the major factors that contributed to the decrease in the nest success, the detailed results of the nest success are shown in Figure 3 and in the Supplementary Table S1. The detailed results for the four breeding stages are also shown in the Supplementary Figures S1–S4.

In all of the breeding sites, the posterior mean values of the nest success after the earthquake were lower than those before earthquake. This is particularly clear for sites No. 10, where the 95% CRI did not overlap with that of the prequake (Fig. 3b, Supplementary Table S1). The site effects, the composite of the effects other than that of the air dose rate, *r*, changed slightly but had no tendency in any particular direction.

The air dose rate had a negative association with the nest success, whereas the site effect also decreased and negatively associated with the nest success in some breeding sites (site Nos. 5, 6, 9, 10, 11). How large was the impact of the site effect when compared with the effect of the air dose rate? The ratios of the effect of the air dose rate and the site effect are shown in Table 6. In the five sites, the effect of the air dose rate was approximately 1.1–8.1 times that of the site effect. The site effect in each of the remaining sites increased and had a positive effect on the nest success. In brief, in all of the breeding sites, the effect of the air dose rate was the major factor associated with the decrease in the nest success.

The next question was the extent to which the air dose rate affected the nest success. Using the posterior mean of the *β*_2_, −3.909 (Table 5), and the posterior distribution of the nest success for site No. 10, where the nest success in the prequake period was the highest among the breeding sites (Fig. 3b, Supplementary Table S1), the maximum drop of the nest success was calculated to be 0.100 with a 0.1 μSv/h increase in the air dose rate (Supplementary Fig. S5).

## Discussion

Our results suggest that the radioactive material emitted by the accidents at the Fukushima Daiichi nuclear power plant negatively affected the reproductive performance of the goshawk. The nest success was negatively associated with the air dose rates measured under the nests. The mechanism was predicted to be external exposure to gamma radiation, but the situation was apparently not so simple. Unless the fallout of radioactive material continues, the air dose rate (the intensity of gamma radiation) decreases over time, and the rates of the breeding stages and the nest success should gradually return to the prequake levels. The rates of the breeding stages and the nest success, however, did not follow this course.

The goshawk is a top predator, and its food organisms occupy a high ecological niche^15^,^16^. It is not clear whether the bioaccumulation of radioactive material would occur in these forested ecosystems, but at least the transfer of radioactive material to higher trophic levels appears to have occurred^17^. In the case of the Chernobyl, the accumulation of ^137^Cs or ^90^Sr was recognised^14^, and a decrease in the nest success of swallows (*Hirundo rustica*) was also reported^5^. Further, it was well investigated that animals at higher trophic levels generally had higher levels of radionuclide concentrations than animals at lower levels^18^,^19^,^20^. Thus, we suspect that the mechanism underlying the decrease in the reproductive success of goshawks was internal exposure by intake of radioactive material emitted by the nuclear accidents at Fukushima, as well as the external exposure from the air dose rates. A high level of air dose rates should reflect the presence of a large amount of radioactive material. If we assume that the goshawks experienced internal exposure, our results can be interpreted as follows: The degree of contamination of the food eaten by the adult birds and the chicks was low in 2011 because it would take time for the radioactive material to prevail or possibly bioaccumulate in the top predators. As time progressed, the contamination reached a top predator, the goshawk, and their reproductive success then decreased.

Some radionuclides, particularly ^90^Sr, are not readily excreted^21^. The negative effects caused by possible continuous internal exposure will therefore be a matter of serious concern. To further identify the effects of internal exposure, the cause of the decline in the goshawk nest success will be clarified by investigating the amounts of radioactive material and the transfer of this material between the goshawks and their prey. Therefore, measuring the accumulation of the radioactive materials in the goshawks and their prey will be determined in future studies.

Accidents at nuclear power plants result in two types of negative effects on reproduction in avian species: direct and indirect effects. Direct effects include the external or internal exposure of the eggs or nestlings as described above. On the other hand, the indirect effects of exposure include the decrease in prey species and the reduction in mean age due to a reduced survival rate^22^ and/or accelerated senescence^23^. With regard to the Goshawk, some studies have reported a reduction in the abundance of prey species near Fukushima^24^,^25^, while the reduction in mean age is thought to be quantifiable as some goshawks are faithful to their chosen nest site where they tend to remain for life^26^. Further field research and DNA analysis is needed to further examine these indirect effects. Young goshawks are assumed to decrease due to the reduction in reproductive performance of the adult goshawks, which then increases the need for their preservation. The existing laws regarding Goshawk preservation requires permission be obtained before even transferring a single feather to other research institution for examination, which will hinder collecting a lot of samples for further research. This situation must first be addressed. If there exists the negative effects of radiation on the reproductive season of the Goshawk, the quantitative or qualitative data (e.g., the nest success rate or the shape of feathers) which will be obtained from surviving birds or other wildlife species may just reflect the individuals that have a relatively lower sensitivity to radiation. In other words, such studies may underestimate the negative influence of radiation. A study plan that enables the monitoring of a broader area over a longer term is also required due to concerns regarding the negative influences on not only wildlife other than the goshawks, but also humans.

## Methods

### Ethic statements

This study was carried out in accordance with the guidelines for the conservation of birds of prey (revised edition) published by the Ministry of the Environment and the guidelines published by the Wildlife Research Center of Kyoto University. All experimental protocols were approved by the Ministry of the Environment.

### Fieldwork

We conducted field research for 22 years. The research period was 1992–2013. The same field method was used over the entire period. We performed a thorough search of the study area to identify the nests of goshawks. We employed a foot survey method^27^ because the study area contained patchy forests in farmlands. We searched all forest areas for alternate nests within 400 m radius from an old nest in each forest patch. For all the nests that were found, we conducted periodic observations, at least once per week, with a 20–40X telescope and 8X binoculars (Nikon, Tokyo, Japan). The investigation period during a year was approximately from the beginning of March, just before the nesting period, to the middle of July, after the fledging of all the nestlings was confirmed. One or two specialists in field research on goshawks conducted the searches and the observations.

The breeding cycle of the goshawk was divided into four stages. The four stages were site occupancy (stage 1), incubating (stage 2), hatching (stage 3), and fledging (stage 4). In each nest, we recorded the success or failure of each of the four stages. In site occupancy (stage 1), an observation of ‘success’ was recorded if we observed an adult goshawk building or refurbishing a nest as evidenced by leafy twigs, or at least occupying the nest area, whereas an observation of ‘failure’ was recorded if not. In incubating (stage 2), a ‘success’ was recorded if we observed from the ground an adult sitting low (incubation posture) in the nest and a ‘failure’ if not. For hatching (stage 3), a ‘success’ was recorded if we observed at least one chick hatched, and a ‘failure’ if no chicks were observed hatched. In fledging (stage 4), a ‘success’ was recorded if we observed at least one fledged young in a tree different than the nest tree, and a ‘failure’ if no fledglings were observed.

On June 28, 2012, we measured the air dose rate of nuclear contaminants under the nests at 13 sites. The 13 sites were chosen at random from 40 nest sites investigated in 2012. The measurements were performed during the daytime, under cloud cover and with very little breeze. Under each nest, we measured the air dose rate three times using a dosimeter (TERRA-P, Sparing-Vist Center, Lviv, Ukraine) and recorded the average reading.

The air dose rate under the nests was considered to have a strong correlation with the total amount of the radioactive materials deposited around the nests and to be appropriate for representing the degree of nuclear contamination of the nest sites. Other alternative doses, such as radiation in nestlings, radiation in feathers, or background radiation in soil, were also important to be measured. However, the level of radiation in nestlings or feathers is speculated to show a different course from the air dose rate due to bioaccumulation. We aimed at identifying the relationship between the degree of nuclear contamination of the nest sites and the reproductive performance of the goshawk; consequently, we thought that the measurement of radiation levels in nestlings or feathers was inappropriate. Moreover, the background radiation in soil was not regarded as appropriate as the measured values were thought to reflect the contamination level of a relatively wider area. The goshawks in our study area inhabited patchy environments which varied widely, partly because of its vicinity to areas of human habitation. In addition, the environment under the nests was not necessarily forested soil. In order to evaluate the association between the direct exposure of the eggs or nestlings and the goshawks’ reproductive success, the measurement value of a smaller, more specific area was required. Therefore, we used the air dose rate under the nests.

### Comparison of pre- and postquake nest success rates

For the four stages of the reproductive cycle of goshawks, we estimated posterior distributions of the rates of site occupancy, incubating, hatching, and fledging before the mega-earthquake, based on a Bayesian method. We used the summed count numbers over 19 years (1992–2010) presented in Table 1; the values occupied against total observed, incubated against occupied, hatched against incubated, and fledged against hatched were used as the observed data.

We assumed that the count numbers for occupied, incubated, hatched, and fledged were binomially distributed. A uniform distribution ranging from 0 to 1 was used as a prior distribution for the Bayesian analysis. We used R 3.0.2 and MCMCpack 1.3–3 for the MCMC sampling. We obtained 10,000 samples and constructed posterior distributions of the four rates.

We calculated the rates of site occupancy, incubating, hatching, and fledging for each year after the earthquake (2011–2013) using the count numbers in Table 2. We mapped these data onto the corresponding posterior distributions.

The nest success was defined as a proportion of fledged nests to incubated nests, based on the definition by Squires & Kennedy^11^. Thus, in this study, the nest success was calculated as fledged/incubated, using the count numbers in Tables 1 and 2. We also constructed a posterior distribution of the nest success before the earthquake and mapped the nest success of each year after the earthquake on it.

### Bayesian estimation of the air dose rate effect and the site effect

For each of the rate of the four breeding stages and the nest success, we performed a hierarchical Bayesian analysis using the 13 sites where the air dose rates were measured. Our model accounted for the differences among the sites. We used recent ten years data because we did not find all the nest sites in our study area until 2004 and there seemed to be little environmental change except for the nuclear accidents during the period. The air dose rate was employed as an explanatory variable; each site was assumed to have a different effect on the rate of the breeding stage or the nest success. We used 0.05 μSv/h as the prequake air dose rate in all the sites because we did not measure the air dose rate under the nests before the earthquake and the value of 0.05 μSV/h was obtained near the measured nest within the same time period^28^ (the average air dose rate for the period April 1, 2010–March 14, 2011). As response variables, occupied against total observed, incubated against occupied, hatched against nested, and fledged against hatched for each site were used for each stage. Fledged against incubated was also used for the analysis of the nest success. We assumed that all variables followed a binomial distribution and designated logit as a link function. Thus, our model was expressed as follows:
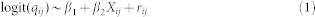

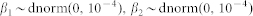

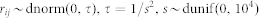
where *i* was the breeding site number (*i* = 1…13), *j* indicated a time period (*j* = 1: before the earthquake, *j* = 2: after the earthquake), *q_ij_* was the rate of the breeding stage or nest success in breeding site *i* in time period *j*, *β*_1_ was the intercept and *β*_2_ was the coefficient of the air dose rate on the rate of the breeding stage or nest success, *X_ij_* was the measured value of the air dose rate under the nest, *r_ij_* was a breeding site effect on the rate of the breeding stage or nest success, dnorm(*μ*, *τ*) was a normal distribution with mean *μ* and precision *τ*, *τ* was a reciprocal of variance, that is, *τ* = 1/*σ*^2^, and dunif(*a*, *b*) was a uniform distribution ranging from *a* to *b*. The intercept *β*_1_ and the coefficient of air dose rate *β*_2_ were assumed to follow a noninformative prior distribution, a normal distribution with mean zero and precision 10^−4^. The breeding site effects *r_ij_* were also assumed to follow a noninformative prior distribution, a normal distribution with mean zero and precision *τ*. *τ* was a hyperparameter of which the value was *τ* = 1/*s*^2^, and *s* followed a uniform distribution ranging from zero to 10^4^, that is, the hyperparameter *τ* was distributed uniformly in the interval of 10^−8^ ≤ *τ* < ∞^29^.

We performed MCMC sampling using JAGS 3.3.0 and rjags 3–12 on R 3.0.2. In the sampling process, the first 10,000 trials were discarded as burn-in, followed by 200,000 trials with a sampling every 100 trials for one MCMC chain. The same process was applied to five chains, and 10,000 samples were generated. We calculated the multivariate version of 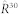
30, a potential scale reduction factor, for the convergence diagnostic and to confirm that the value was very close to 1.0.

We constructed posterior distributions of the coefficient of the air dose rate *β*_2_, the rate of the breeding stage or the nest success *q_ij_*, and the breeding site effect *r_ij_*, using the obtained 10,000 samples. In addition, we calculated the effect due to the difference of the air dose rate, 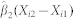, and the effect due to the difference of the breeding site effect, 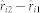, and we compared them to determine which effect had more influence on the change of the nest success. 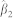, 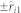, and 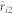 were posterior mean values.

## Author Contributions

K.E. conceived and designed the study, managed the project. K.E., R.H. and K.M. performed the fieldwork. R.H. managed the data. K.M. and J.M. analysed the data and developed the figures. K.M. drafted the initial manuscript. All the coauthors commented on and provided substantial edits to the manuscript.

## Supplementary Material

Supplementary Information Supplementary Information

## Figures and Tables

**Table 1:** Prequake reproductive performance of goshawks in 1992–2010

Year	Total observed	Occupied	Incubated	Hatched	Fledged
1992	20	19	19	16	13
1993	26	24	23	19	18
1994	30	29	21	18	16
1995	31	28	26	23	22
1996	28	26	22	20	19
1997	33	19	19	16	14
1998	35	28	19	19	18
1999	34	24	24	21	18
2000	39	25	21	18	16
2001	37	28	25	21	20
2002	36	27	26	23	21
2003	37	30	29	25	21
2004	40	29	25	23	18
2005	44	31	26	22	19
2006	40	24	22	21	19
2007	41	28	25	22	18
2008	46	28	24	21	19
2009	43	25	21	19	17
2010	44	23	22	19	16
Total	684	495	439	386	342

**Table 2:** Postquake reproductive performance of goshawks in 2011–2013

Year	Total observed	Occupied	Incubated	Hatched	Fledged
2011	45	22	20	18	15
2012	40	25	20	17	11
2013	37	22	18	12	9
Total	122	69	58	47	35

**Table 3:** Pre- and postquake reproductive performance of goshawks at 13 breeding sites in recent ten years and the postquake air dose rate. The air dose rates were measured on June 28, 2012

	2004–2010					2011–2013					
Site No.	Total observed	Occupied	Incubated	Hatched	Fledged	Total observed	Occupied	Incubated	Hatched	Fledged	Air dose rate (uSv/h)
1	7	7	7	7	5	3	2	1	0	0	0.85
2	1	1	1	1	1	3	3	3	3	3	0.29
3	6	5	5	4	4	3	3	3	2	1	0.73
4	7	7	7	6	5	3	3	3	2	2	0.40
5	7	3	3	3	3	3	3	3	0	0	0.28
6	6	5	5	5	4	3	3	3	2	1	0.27
7	6	6	6	6	4	3	3	3	3	2	0.30
8	6	5	3	2	1	3	3	2	2	2	0.27
9	6	6	6	5	5	3	3	3	2	1	0.44
10	7	7	7	7	7	3	2	2	2	0	0.61
11	4	4	3	3	3	3	3	3	1	1	0.36
12	6	6	6	5	5	3	3	3	3	3	0.49
13	6	5	5	4	4	3	2	2	2	1	0.53
Total	75	67	64	58	51	39	36	34	24	17	

**Table 4:** Statistics of the rates of the four breeding stages and the nest success in pre- and postquake years. The rates in each postquake year are compared with the 95% credible intervals (CRI) of the prequake years' rates. The position of the rate of each postquake year in the corresponding 95% CRI is shown in the percentile columns. The rates outside the 95% CRIs are denoted by *

	1992–2010			2011		2012		2013	
	2.5%	Mean	97.5%	Rate	Percentile	Rate	Percentile	Rate	Percentile
Each breeding stage:									
(Stage 1) Site occupancy	0.6887	0.7230	0.7556	0.4889	0.01%*	0.6250	0.01%*	0.5946	0.01%*
(Stage 2) Incubating	0.8549	0.8851	0.9114	0.9091	96.02%	0.8000	0.01%*	0.8182	0.01%*
(Stage 3) Hatching	0.8456	0.8775	0.9065	0.9000	93.24%	0.8500	4.38%	0.6667	0.01%*
(Stage 4) Fledging	0.8505	0.8839	0.9138	0.8333	0.26%*	0.6471	0.01%*	0.7500	0.01%*
Nest success									
Stage 3–4	0.7375	0.7779	0.8149	0.7500	8.37%	0.5500	0.01%*	0.5000	0.01%*

**Table 5:** Statistics of the coefficients of the air dose rate. The 95% CRIs of the air dose rate coefficients, *β*_2_, are shown for the four breeding stages and the nest success. The proportions of *β*_2_ with negative values in the posterior distributions are also shown in the *β*_2_ < 0 column and are denoted by * if they exceed 97.5%

	2.5%	Mean	97.5%	*β*_2_ < 0
Each breeding stage:				
(Stage 1) Site occupancy	−6.604	−1.268	3.550	0.7127
(Stage 2) Incubating	−49.913	−5.080	28.973	0.6934
(Stage 3) Hatching	−7.417	−3.181	0.321	0.9623
(Stage 4) Fledging	−8.921	−4.114	−0.393	0.9840*
Nest success				
Stage 3–4	−6.888	−3.909	−1.283	0.9991*

**Table 6:** Air dose rate effect vs. breeding site effect. The air dose rate effect and the breeding site effect on nest success are compared. For each breeding site, the effect due to the difference of the air dose rate (Dose effect) and the effect due to the difference of the breeding site effect (Site effect), between pre- and postquake years, are shown. The Dose effect was calculated as 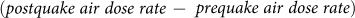
where −3.909 is the posterior mean of *β*_2_, the coefficient of the air dose rate. The Site effect was calculated as 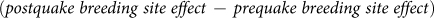
The ratio of the Dose effect to the Site effect is shown in the D/S column. If the Site effect takes positive value, it is denoted by -, for we focused attention on the negative effects on the nest success. A ratio higher than 1.0 indicates a greater importance of the Dose effect

Site No.	Dose effect	Site effect	D/S
1	−3.127	0.061	-
2	−0.938	0.284	-
3	−2.658	0.085	-
4	−1.368	0.308	-
5	−0.899	−0.818	1.099
6	−0.860	−0.268	3.212
7	−0.977	0.267	-
8	−0.860	0.745	-
9	−1.525	−0.189	8.087
10	−2.189	−0.662	3.305
11	−1.212	−0.444	2.731
12	−1.720	0.496	-
13	−1.876	0.069	-

## References

[b1] Ozaki K. *et al.* A mechanistic approach to evaluation of umbrella species as conservation surrogates. Conserv. Biol. 20, 1507–1515 (2006). 17002768 10.1111/j.1523-1739.2006.00444.x

[b2] Sergio F. *et al.* Top predators as conservation tools: ecological rationale, assumptions, and efficacy. Annu. Rev. Ecol. Evol. Syst. 39, 1–19 (2008).

[b3] DeSante D. F. & Geupel G. R. Landbird productivity in central coastal California: the relationship to annual rainfall, and a reproductive failure in 1986. Condor 89, 636–653 (1987).

[b4] Ellegren H., Lindgren G., Primmer C. R. & Møller A. P. Fitness loss and germline mutations in barn swallows breeding in Chernobyl. Nature 389, 593–596 (1997). 9335497 10.1038/39303

[b5] Møller A. P. *et al.* Condition, reproduction and survival of barn swallows from Chernobyl. J. Anim. Ecol. 74, 1102–1111 (2005).

[b6] Møller A. P. & Mousseau T. A. Biological consequences of Chernobyl: 20 years on. Trends Ecol. Evol. 21, 200–207 (2006). 16701086 10.1016/j.tree.2006.01.008

[b7] Stephan V. Chernobyl: Poverty and stress pose ‘bigger threat’ than radiation. Nature 437, 181–181 (2005). 16148902 10.1038/437181b

[b8] Mousseau T. A., Nelson N. & Shestopalov V. Don't underestimate the death rate from Chernobyl. Nature 437, 1089–1089 (2005). 16237420 10.1038/4371089a

[b9] Møller A. P., Karadas F. & Mousseau T. A. Antioxidants in eggs of great tits *Parus major* from Chernobyl and hatching success. J. Comp. Physiol. B 178, 735–743 (2008). 18392836 10.1007/s00360-008-0262-z

[b10] Møller A. P., Mousseau T. A., Lynn C., Ostermiller S. & Rudolfsen G. Impaired swimming behaviour and morphology of sperm from barn swallows *Hirundo rustica* in Chernobyl. Mutat. Res. Genet. Toxicol. Environ. Mutagen. 650, 210–216 (2008). 10.1016/j.mrgentox.2007.12.006 18218334

[b11] Squires J. R. & Kennedy P. L. Northern Goshawk ecology: an assessment of current knowledge and information needs for conservation and management. Stud. Avian Biol. 31, 8–62 (2006).

[b12] Horie R., Endo K., Nonaka J. & Ozaki K. Seasonal change in home range of male Northern Goshawk in central Japan. Jpn. J. Ornithol. 56, 22–32 (2007).

[b13] Nuclear Regulation Authority. 2012. Available: Analysis Results Concerning (i) Gamma-emitting Nuclidesand (ii) Sr-89 and Sr-90 (Second Distribution Survey) by MEXT http://radioactivity.nsr.go.jp/en/contents/6000/5636/24/338_Sr_0912018_e.pdf Accessed 11 August, 2014.

[b14] Yablokov A. V., Nesterenko V. B. & Nesterenko A. V. Chernobyl: Consequences of the catastrophe for people and the environment. (Blackwell, Boston, 2010). 20052781

[b15] Kenward R. The Goshawk. (T&A D Poyser, London, 2006).

[b16] Ozaki K. & Endo K. (eds) Ecology and conservation of Northern Goshawk: toward its population conservation. (Japan forest technology association, Tokyo, 2008) (in Japanese).

[b17] Murakami M. *et al.* Biological proliferation of cesium-137 through the detrital food chain in a forest ecosystem in Japan. Sci. Rep. 4, 3599 (2014). 24398571 10.1038/srep03599 PMC3884222

[b18] Kryshev I. I., & Ryabov I. N. About the efficiency of trophic levels in the accumulation of Cs-137 in fish of the Chernobyl NPP cooling pond. Biological and radioecological aspects of the consequences of the Chernobyl Accident. 116–121 (USSR Academy of Sciences, Moscow, 1990).

[b19] Kryshev I., Alexakhin R. & Makhonko K. Radioecological consequences of the Chernobyl accident. (Nuclear Society, Moscow, 1992).

[b20] Smith M. H., Oleksyk T. K., & Tsyusko O. Effects of trophic position and ecosystem type on the form of the frequency distribution of radiocesium at Chornobyl and nuclear sites in the United States. In:: Proceedings of the international symposium: transfer of radionuclides in biosphere: prediction and assessment, December 18–19, 2002. 37–48 (Mito, Japan, 2002).

[b21] Froidevaux P., Bochud F. & Haldimann M. Retention half times in the skeleton of plutonium and ^90^Sr from above-ground nuclear tests: A retrospective study of the Swiss population. Chemosphere 80, 519–524 (2010). 20466404 10.1016/j.chemosphere.2010.04.049

[b22] Møller A. P., Bonisoli-Alquati A., Rudolfsen G., & Mousseau T. A. Elevated mortality among birds in Chernobyl as judged from skewed age and sex ratios. PLoS ONE 7, e35223 (2012). 22514722 10.1371/journal.pone.0035223 PMC3324427

[b23] Møller A. P. & De Lope F. Senescence in a short-lived migratory bird: age-dependent morphology, migration, reproduction and parasitism. J. Anim. Ecol. 68, 163–171 (1999).

[b24] Møller A. P. *et al.* Abundance of birds in Fukushima as judged from Chernobyl. Environ. Pollut. 164, 36–39 (2012). 22321986 10.1016/j.envpol.2012.01.008

[b25] Møller A. P., Nishiumi I., Suzuki H., Ueda K., & Mousseau T. A. Differences in effects of radiation on abundance of animals in Fukushima and Chernobyl. Ecol. Indic. 24, 75–81 (2013).

[b26] Møller A. P. & Nielsen J. T. Parental defense of offspring and life history of a long-lived raptor. Behav. Ecol. 25, 1505–1512 (2014).

[b27] Kennedy P. L. & Andersen D. E. Research and Monitoring Plan for Northern Goshawks (Accipter Gentilis Atricapillus) in the Western Great Lakes Region. (Minnesota Cooperative Fish and Wildlife Research Unit, University of Minnesota, 2005).

[b28] Nuclear Regulation Authority. Environmental Radioactivity and Radiation Survey Report (fiscal 2010–2011) http://www.kankyo-hoshano.go.jp/en/01/0101flash/01012009.html Accessed 15 August, 2014.

[b29] Gelman A. Prior distributions for variance parameters in hierarchical models. Bayesian Anal. 1, 515–533 (2006).

[b30] Brooks S. P. & Gelman A. General methods for monitoring convergence of iterative simulations. J. Comput. Graph. Stat. 7, 434–455 (1998).

