# Supplementary material for: Effects of the Fukushima Daiichi nuclear accident on goshawk reproduction
Source: Sci Rep. 2015 Mar 24;5:9405. doi: 10.1038/srep09405 (PMC4371089; doi:10.1038/srep09405)
Supplement: Supplementary Information [file srep09405-s1.pdf]

[Supplementary Information]

Effects of the Fukushima Daiichi nuclear accident on goshawk reproduction

Kaori Murase, Joe Murase, Reiko Horie & Koichi Endo

Supplementary Table S1

Supplementary Figure S1–S5

Supplementary Equation S1

Supplementary Table S1 | Statistics of the results of the Bayesian analysis for the nest success.

This table shows the statistics for Figure 3. The 95% CRIs of the parameters of the Bayesian model for the nest success rare shown. **a,** The air dose rate coefficient, β_2. The proportion of β_2 showing a negative value is indicated in the $\beta_2 < 0$ column. **b,** The nest success and the breeding site effects (pre- and postquake) for each breeding site. The q (pre) and q (post) columns indicate the nest success of pre- and postquake years, respectively. The r (pre) and r (post) columns indicate the breeding site effects of pre- and postquake years, respectively.

a

	2.5%	Mean	97.5%	$\beta_2 < 0$
β_2	-6.888	-3.909	-1.283	0.9991

b

Site No.	q (pre)			q (post)			r (pre)			r (post)		
	2.5%	Mean	97.5%	2.5%	Mean	97.5%	2.5%	Mean	97.5%	2.5%	Mean	97.5%
1	0.5488	0.7738	0.9194	0.0138	0.1924	0.5725	-1.481	-0.154	0.864	-1.757	-0.093	1.263
2	0.5496	0.8031	0.9682	0.4468	0.6889	0.9459	-1.217	0.099	1.701	-0.654	0.383	2.245
3	0.5743	0.7951	0.9465	0.0539	0.2747	0.6210	-1.243	0.000	1.213	-1.191	0.085	1.475
4	0.5419	0.7728	0.9193	0.2737	0.5531	0.8454	-1.460	-0.160	0.852	-0.939	0.148	1.579
5	0.6199	0.8214	0.9737	0.1073	0.5024	0.7508	-0.905	0.225	1.871	-2.740	-0.593	0.413
6	0.5753	0.7938	0.9435	0.2346	0.5756	0.8067	-1.225	-0.013	1.176	-1.836	-0.281	0.726
7	0.5087	0.7623	0.9126	0.3296	0.6196	0.8676	-1.564	-0.219	0.773	-1.173	0.048	1.372
8	0.3432	0.7143	0.8962	0.4311	0.6857	0.9436	-2.320	-0.462	0.552	-0.800	0.283	2.107
9	0.6023	0.8026	0.9493	0.1578	0.4550	0.7346	-1.096	0.047	1.294	-1.551	-0.142	1.008
10	0.6776	0.8434	0.9789	0.0464	0.3013	0.6235	-0.565	0.401	2.161	-2.066	-0.262	0.912
11	0.6146	0.8216	0.9763	0.1969	0.5112	0.7712	-0.933	0.235	1.991	-1.656	-0.209	0.885
12	0.5989	0.8028	0.9484	0.2661	0.5549	0.9143	-1.113	0.047	1.265	-0.453	0.543	2.599
13	0.5758	0.7950	0.9455	0.1331	0.4223	0.7689	-1.209	-0.003	1.227	-1.223	0.066	1.464

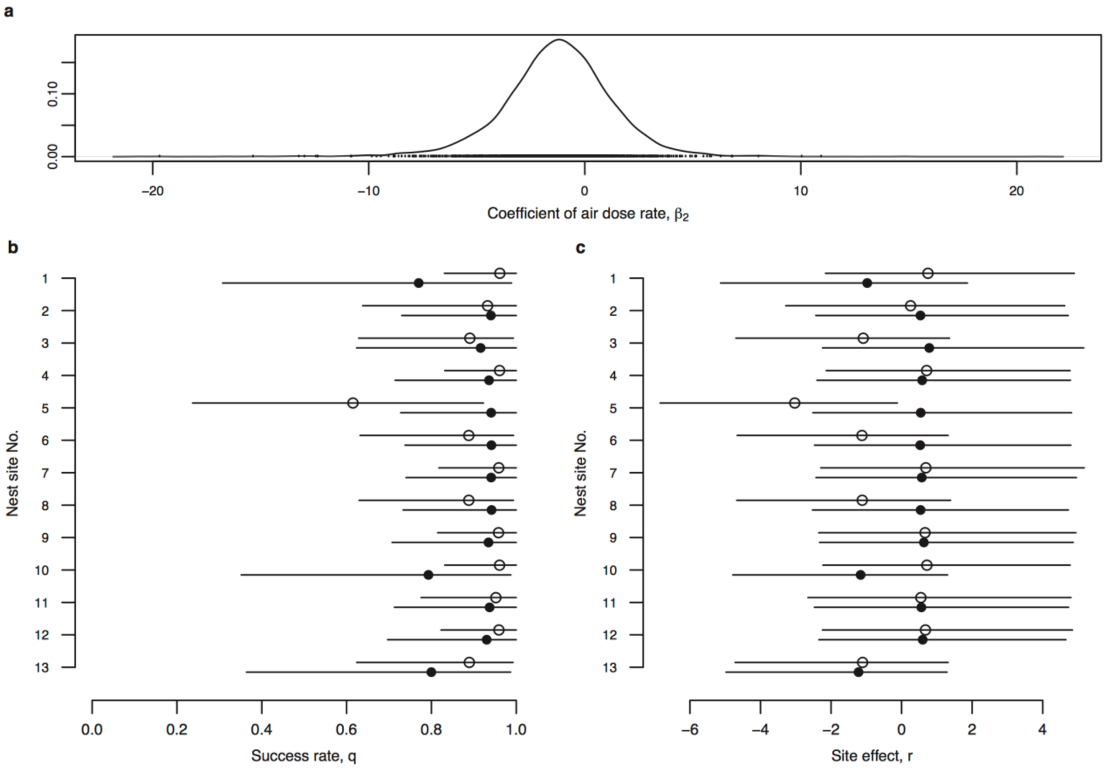

Supplementary Figure S1 | Results of the Bayesian analysis for site occupancy (stage 1).

The posterior distributions of the parameters of the hierarchical Bayesian model for the site occupancy rate are shown. **a,** The air dose rate coefficient, β_2. This is the same figure as Figure 2a, right, except for its range. **b,** The site occupancy rate of each breeding site. **c,** Site effect of each breeding site. The open circles represent prequake years, and the filled circles represent postquake years. The horizontal bars indicate 95% CRIs.

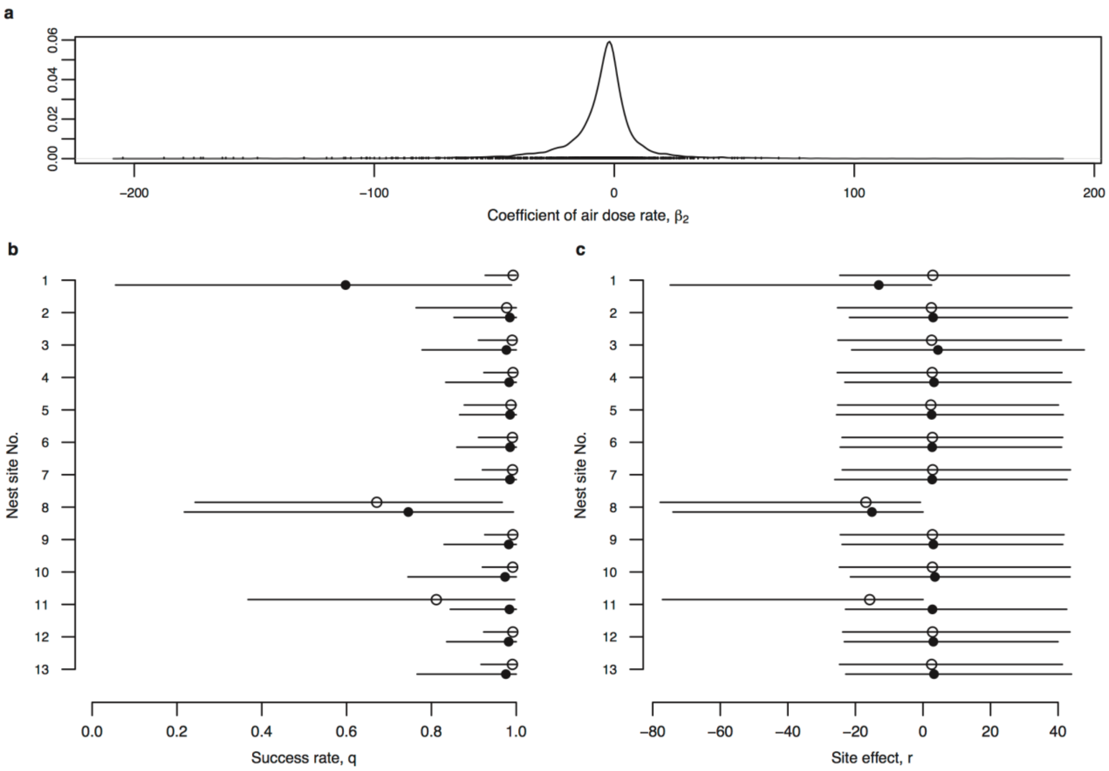

Supplementary Figure S2 | Results of the Bayesian analysis for incubating (stage 2). The posterior distributions of the parameters of the hierarchical Bayesian model for the incubating rate are shown. **a,** The air dose rate coefficient, β_2. This is the same figure as Figure 2b, right, except for its range. **b,** The incubating rate of each breeding site. **c,** Site effect of each breeding site. The open circles represent prequake years, and the filled circles represent postquake years. The horizontal bars indicate 95% CRIs.

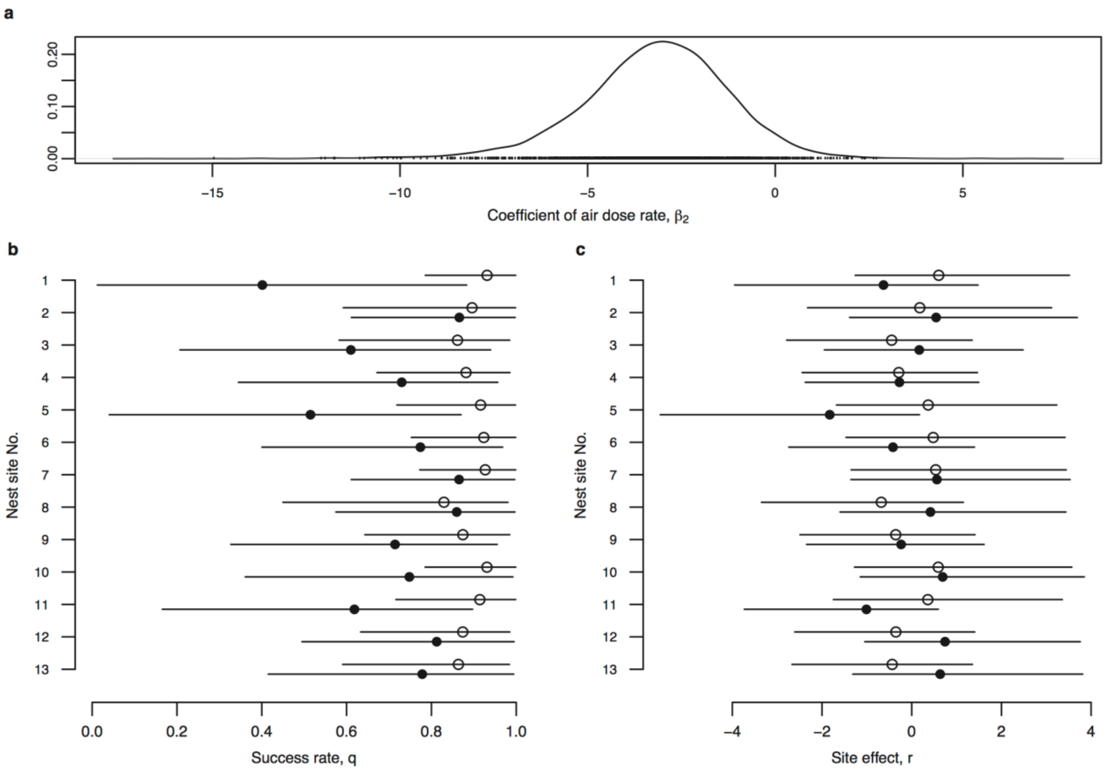

Supplementary Figure S3 | Results of the Bayesian analysis for hatching (stage 3). The posterior distributions of the parameters of the hierarchical Bayesian model for the hatching rate are shown. **a,** The air dose rate coefficient, β_2. This is the same figure as Figure 2c, right, except for its range. **b,** The hatching rate of each breeding site. **c,** Site effect of each breeding site. The open circles represent prequake years, and the filled circles represent postquake years. The horizontal bars indicate 95% CRIs.

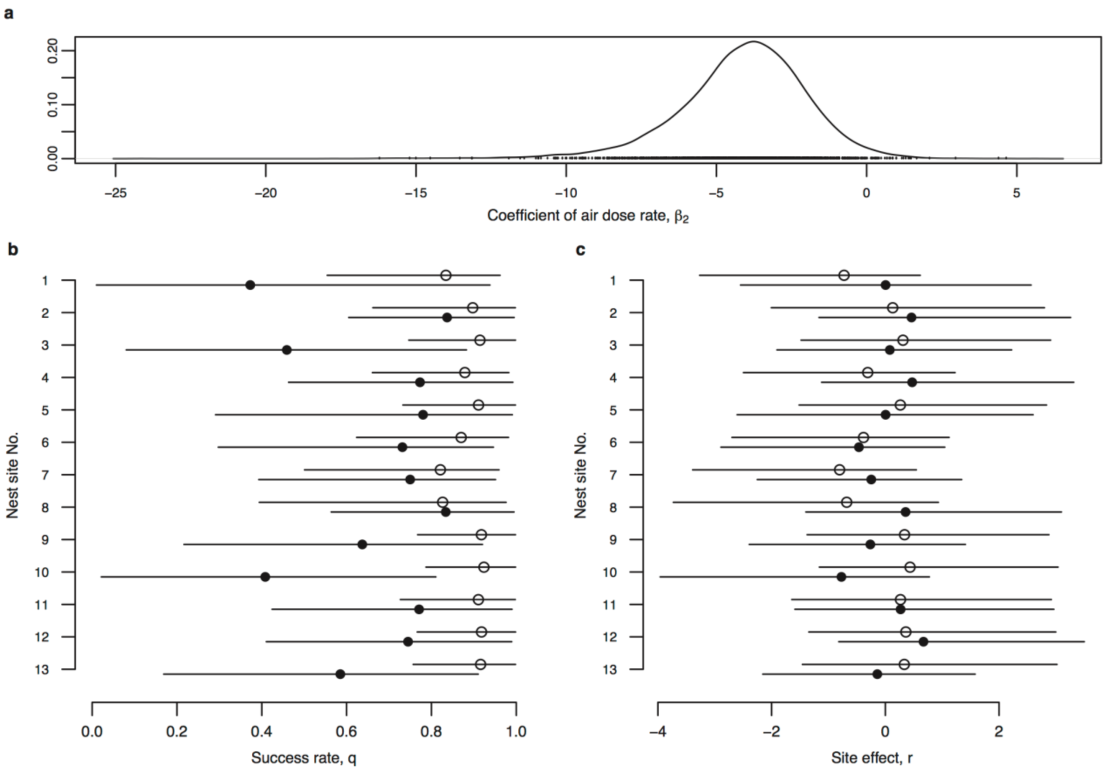

Supplementary Figure S4 | Results of the Bayesian analysis for fledging (stage 4). The posterior distributions of the parameters of the hierarchical Bayesian model for the fledging rate are shown. **a,** The air dose rate coefficient, β_2. This is the same figure as Figure 2d, right, except for its range. **b,** The fledging rate of each breeding site. **c,** Site effect of each breeding site. The open circles represent prequake years, and the filled circles represent postquake years. The horizontal bars indicate 95% CRIs.

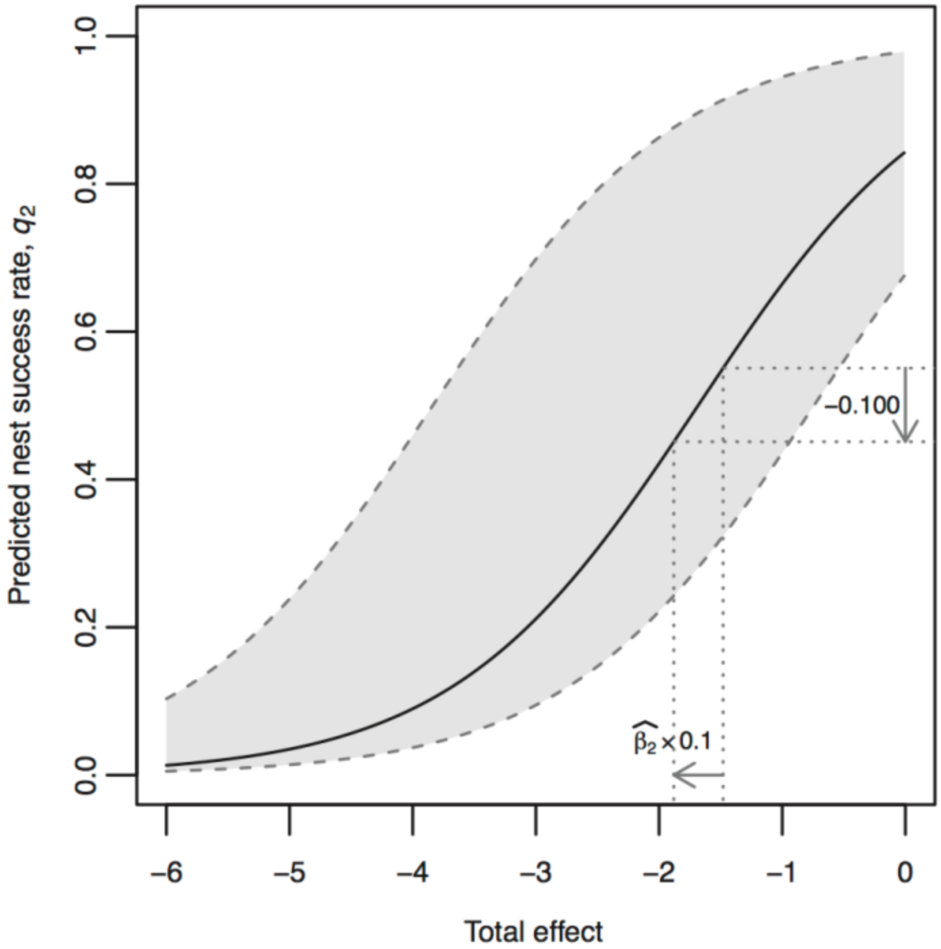

Supplementary Figure S5 | Relationship between air dose rate and nest success.

The relationship between the total effect and the nest success is shown. The total effect equals the sum of the effects of the differences in the air dose rates and the site effect differences between pre- and postquake years. The grey-filled region indicates the 95% CRI of a predicted distribution of the nest success. The 95% CRI of the nest success of the breeding site No.10 was employed as a baseline (i.e., total effect = 0) because the nest success in the prequake years was the highest, and the profound difference is appropriate for showing the relationship between the total effect and the nest success. The upper and lower dashed lines indicate the 97.5% and 2.5% percentiles of the predicted nest success, respectively. The solid line indicates the mean. If there was a 0.1 μSv/h increase in the air dose rate (corresponding to a -0.391 decrease in total effect when the posterior mean of β_2, -3.909, is used) and the total effect fell below approximately -2.0, a possible decrease in the nest success due to the air dose rate would be no greater than 0.100.

This prediction was made as

$$\tilde{q}_2 = \frac{\frac{\hat{q}_1}{1-\hat{q}_1}\exp(x)}{1+\frac{\hat{q}_1}{1-\hat{q}_1}\exp(x)} \qquad \text{(S1)},$$

where $\hat{q}_1$ is the baseline value of the nest success, $\tilde{q}_2$ is the predicted nest success, and x is the total effect value. The total effect value, x, is calculated as

$$\hat{\beta}_2 \times (\text{change of air dose rate}) + (\text{change of site effect}),$$

where $\hat{\beta}_2$ is assumed to be -3.909 (Table 2b or Supplementary Table 1a).

Supplementary Equation 1 is derived as follows:
Because we assumed that $\hat{q}_1$ and $\tilde{q}_2$ followed binomial distributions and built a model as Equation 1, we obtain

$$\text{odds1} = \hat{q}_1/1-\hat{q}_1 = \exp(\hat{\beta}_1 + \hat{\beta}_2 \dot{X}_{i,1} + \hat{r}_{i,1}), \text{odds2} = \tilde{q}_2/1-\tilde{q}_2 = \exp(\hat{\beta}_1 + \hat{\beta}_2 X_{i,2} + r_{i,2}),$$

then,

$$\frac{\text{odds2}}{\text{odds1}} = \frac{\exp(\hat{\beta}_1 + \hat{\beta}_2 X_{i,2} + r_{i,2})}{\exp(\hat{\beta}_1 + \hat{\beta}_2 \dot{X}_{i,1} + \hat{r}_{i,1})} = \exp\left\{\hat{\beta}_2 (X_{i,2} - \dot{X}_{i,1}) + (r_{i,2} - \hat{r}_{i,1})\right\},$$

where $\dot{X}_{i,1}$ is a measurement of air dose rate in prequake years (i.e., 0.05 μSv/h).

Let us define x as

$$x = \hat{\beta}_2 (X_{i,2} - \dot{X}_{i,1}) + (r_{i,2} - \hat{r}_{i,1}).$$

Then,

$$\frac{\text{odds2}}{\text{odds1}} = \exp(x) \Leftrightarrow \frac{\tilde{q}_2}{1-\tilde{q}_2} = \text{odds1} \cdot \exp(x) \Leftrightarrow \tilde{q}_2 = \frac{\text{odds1} \cdot \exp(x)}{1 + \text{odds1} \cdot \exp(\text{x})}.$$

Thus, we obtain Supplementary Equation S1.
